# A new book “Rare Diseases: Integrative PPPM Approach as the Medicine of the Future”

**DOI:** 10.1186/1878-5085-5-S1-A16

**Published:** 2014-02-11

**Authors:** Meral Özgüç

**Affiliations:** 1 DNA/ Cell Bank for Rare Diseases, Hacettepe University, Faculty of Medicine, Ankara, Turkey

## 

Rare diseases (RDs) or orphan diseases are a group of diseases with a low prevalence (less than 5 persons per 10.000 individuals). There are about 1000 currently recognised RDs from assumed 6000-8000 ones. Almost 80% of RDs have a genetic origin with symptoms appearing in prenatal and early postnatal periods. Due to the wide spectrum of RDs and a lack of sufficient knowledge about individual RDs, the correct diagnosis is difficult to make. Furthermore, currently there are no appropriate treatment approaches for most of the RDs. Due to the molecular background of most RD pathologies, it is expected that the multimodal approach (*omics, pharmacogenetics, medical imaging, etc.) with multidisciplinary professionals should be instrumental for the ‘‘personalisation’’ to diagnose individual RDs, to create effective preventive measures and to develop targeted therapies. Recent achievements in bio/medical sciences let us trust in a prompt translation of innovative technologies into daily clinical practice. In order to increase the impact of personalized medicine, education at the level of all stakeholders is needed. One aspect that should be taken into consideration is the very fast advances in the technologies that are used and the information dissemination that is lagging behind. The stakeholders can be numerous in the area of health care and education and awareness raising should be done at a wide spectrum such as general public, policy makers, nurses, genetic counsellers, ethics review committees. Within the concept for education for all, I would also like to draw the attention to the technological gap that exists between countries that innovate and develop the technologies and the less developed countries where the technology transfer is at a high cost and human capabilities, critical mass and the physical infrastructure is not sufficient. Looking towards Horizon 2020, it may be a good opportunity to take up the area of education and information dissemination as one of the goals of the new program. A global approach to form networks between developed and less developed countries will definitely benefit all patients in the long run. The European Association for Predictive, Preventive and Personalised Medicine (EPMA) actively promotes the scientific and technological efforts, expert recommendations and creation of new guidelines in the field of RD healthcare. This initiative has been triggered through The EPMA Journal launched in 2010 as the professional forum in PPPM. The new book “Rare Diseases: Integrative PPPM Approach as the Medicine of the Future” is dedicated to all aspect related to the prediction, prevention and personalised treatments of RDs. This volume is intended to serve as a reference source for scientific and medical centres involved in the field with a special emphasis on healthcare promotion and innovations intended to combat RDs. This book can be used as education material; it has a wide range of chapters from emerging diagnostic methods, biobanking, iPScell technologies to genetic therapies. Hopefully it can also find high circulation in developing countries to be used as lecture material in graduate and postgraduate curricula.

6th European Conference on Rare Diseases & Orphan Products, 23-25 May 2012, Brussels, Executive Summary, EURORDIS

Communication from the Commission to the European Parliament, The Council, the European Economic and Social Committee and the Committee of the Regions, “on Rare Diseases: Europe's challenges”, Commission of the European Communities, Brussels, 2008.

Omics in Personalised Medicine, Summary Report, Workshop to explore the role of -omics in the development of personalised medicine European Commission, DG Research - Brussels, 29-30 April 2010
